# Sporadic Case of CHARGE Syndrome With Chromodomain-Helicase-DNA-Binding Protein 7 (CDH7) Gene Mutation

**DOI:** 10.7759/cureus.12291

**Published:** 2020-12-26

**Authors:** Batool Wael Alnahar, Ahmed M Alsheikh, Amani G Alruhaimi, Ibtesam A Abdulghani

**Affiliations:** 1 Medicine, College of Medicine, Almaarefa University, Riyadh, SAU; 2 Physiotherapy, Dr. Abdul Rahman Al Mishari Hospital, Riyadh, SAU; 3 Pediatrics, Dr. Abdul Rahman Al Mishari Hospital, Riyadh, SAU

**Keywords:** charge syndrome, cdh7, mutation, congenital anomalies, sporadic, multidisciplinary

## Abstract

CHARGE syndrome with chromodomain-helicase-DNA-binding protein 7 (CDH7) gene mutation is a genetic disease with an autosomal dominant gene. This syndrome involves a combination of six congenital anomalies (heart anomalies, coloboma of the eye, retardation of the growth or development, atresia of the choana, ear anomalies, and genital anomalies). Here, we present a case of a 15-month-old male child who was born to a 23-year-old healthy mother with no history of any exposure to teratogenic materials or drugs. The patient was delivered by cesarean section because of the failure of progression at 39 weeks of pregnancy with several health problems that started with the respiratory system right after birth. On examination, he was found to be suffering from several congenital anomalies, including heart, face, eyes, ears, and genitalia. A genetic analysis was performed for the patient, and a mutation in the CDH7 gene was found. The patient was diagnosed as a sporadic case of CHARGE syndrome. The patient's treatment plan is a multidisciplinary team effort to alleviate his quality of life and further increase life expectancy.

## Introduction

CHARGE syndrome was first described in 1979 by Hall B and Hittner HM [1-2]. It is a genetic disorder with an autosomal dominant inheritance pattern [3]. It consists of six features of physical sign-related congenital anomalies, which include heart anomalies, coloboma of the eye, retardation of growth or development, atresia of the choana, ear anomalies, and genital anomalies [4]. For the diagnosis of CHARGE syndrome, the presence of four out of the six anomalies are necessary to confirm the diagnosis and is confirmed by genetic sequencing [5]. Furthermore, the incidence of this syndrome ranges from 0.1 to 1 per 10,000 live births [5]. The prevalence rate of CHARGE syndrome was reported in the range of 1:8500 in Europe and 1:12000 in Canada [6]. Here, we present a case of sporadic CHARGE syndrome with chromodomain-helicase-DNA-binding protein 7 (CDH7) gene mutation.

## Case presentation

This is a case of a 15-month-old male who was referred to King Saud Medical Complex in Riyadh, Saudi Arabia, for a multidisciplinary follow-up plan and genetic testing. Regarding his history, the patient was delivered at the 39^th^ gestational week of a 23-year-old mother by cesarean section due to the failure of progression, weighing 2.4 kg, and was reported to have developed respiratory distress upon birth. Regarding the mother, she is medically free and was on multivitamins while there were no reports of any radiation or teratogenic exposure that was reported during her pregnancy. After birth, the patient was placed in the neonatal intensive care unit (NICU) for six months due to his abnormal respiration. There is no family history of any genetic disorders, consanguinity, or congenital anomalies.

**Figure 1:**
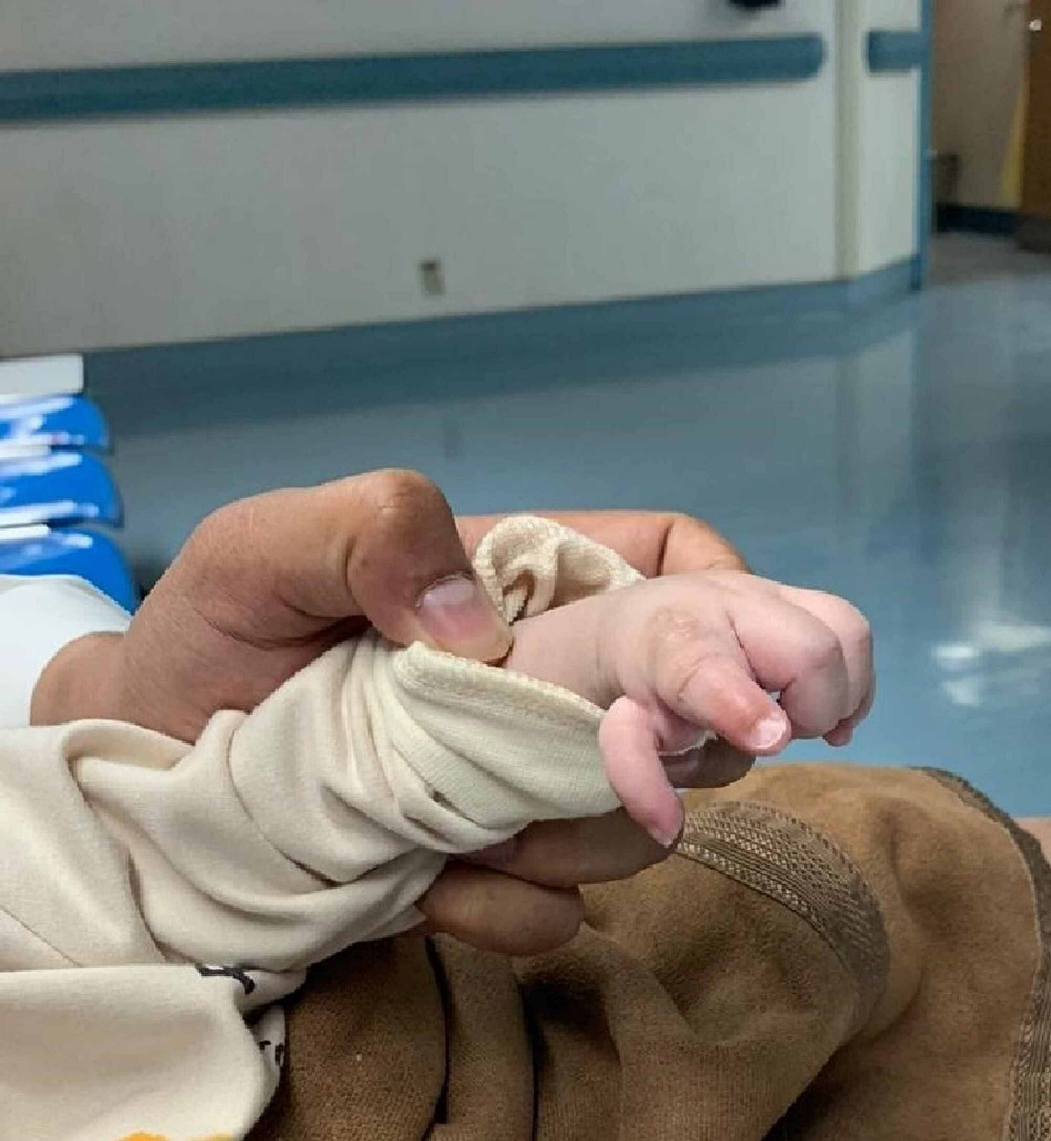
Hypo-plastic left thumb

Initially, the patient was suffering from developmental delay and extensor muscle weakness of the upper and lower limbs. Moreover, an epiglottopexy procedure was performed due to choanal atresia and a high arched palate. Afterward, he was breathing on room air through a nasal cannula. Ever since the epiglottopexy, the patient has been on continuous positive airway pressure (CPAP) with no attempt to wean him off the ventilation due to poor response. Even though the patient did not present any gastrointestinal abnormalities, there was a difficulty in feeding, which led to nasogastric tube insertion for oral nutrition and supplementation. The patient also developed hypogonadotropic hypogonadism, which was confirmed by testing; an abnormal facial shape, which was a dysmorphic triangular face; coloboma; and bilateral low-set ears. A shallow acetabular roof, micropenis, small bilateral kidney, high arched palate, and hypo-plastic left thumb was also noted (Figure 1). Regarding the cardiovascular system, the patient had a moderate to large ventricular septal defect (VSD), dilated left atrium and left ventricle, and mild pulmonary stenosis. Furthermore, the patient did not develop any seizures or loss of consciousness, but he was hypotonic. However, his gastrointestinal system showed no abnormalities.

**Figure 2:**
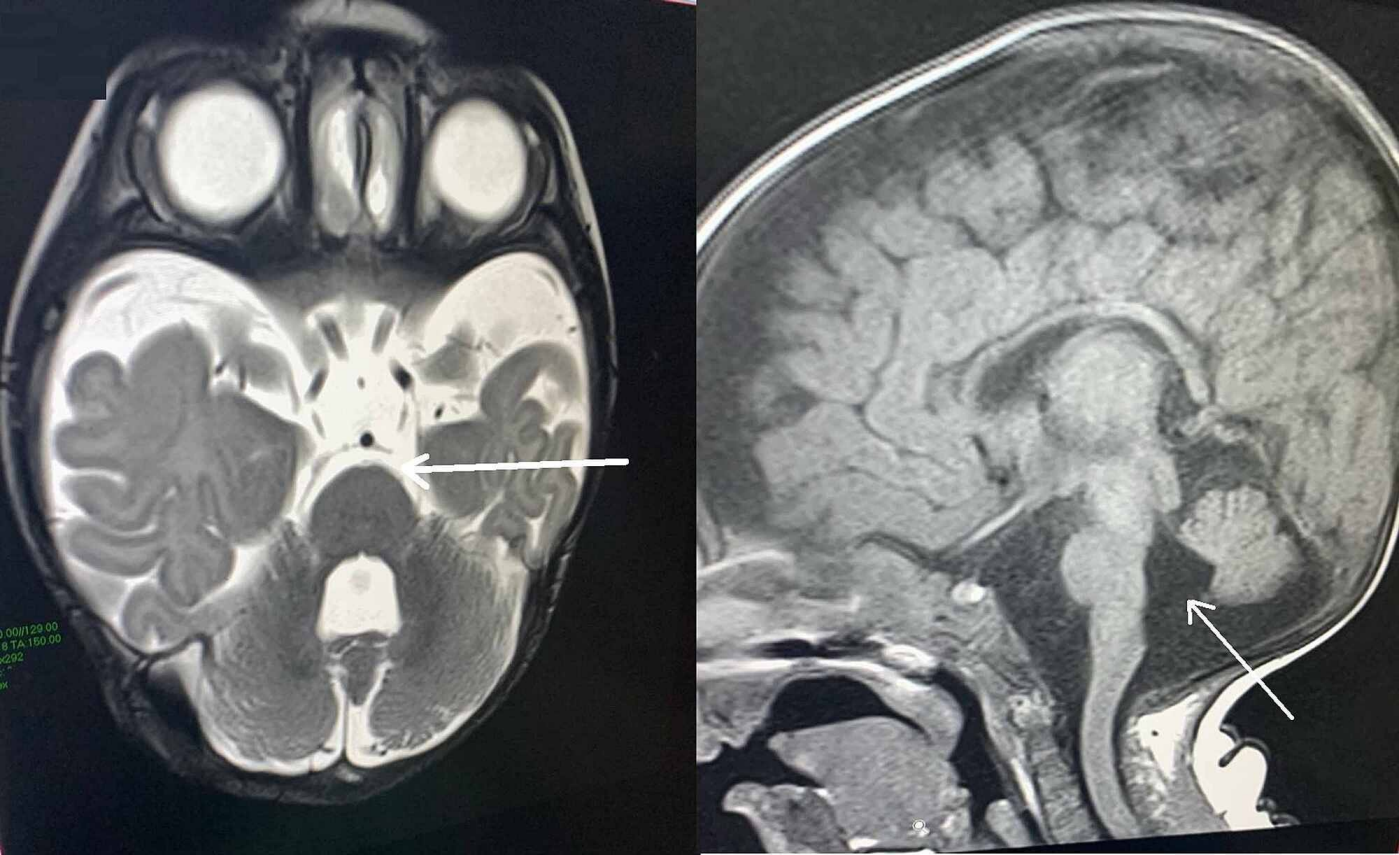
Magnetic resonance imaging of the brain showing mild periventricular leukomalacia, benign external hydrocephalus with the suggestion of Blake's pouch cyst Arrows showing Blake's pouch cyst

Regarding the investigation, his blood work was within normal limits except for a low serum creatinine of 13 µmol/L (normal range: 71-133 µmol/L) and hyponatremia of 130 mEq/L. Meanwhile, his brain magnetic resonance imaging (MRI) showed mild periventricular leukomalacia, benign external hydrocephalus, with the suggestion of Blake's pouch cyst (Figure 2). The genetic investigation was performed for the patient, and he was found to have chromodomain helicase DNA-binding protein 7 (CHD7), c.2572C˃T p(Arg858), and the diagnosis of CHARGE syndrome was confirmed based on the physical examination and the gene sequencing report. The patient is in hospital and is followed up by a multidisciplinary team consisting of a cardiologist, neurologist, nephrologist, general pediatrician, gastroenterologist, otolaryngologist, ophthalmologist, pulmonologist, and physiotherapist with a plan to try and increase and improve his life expectancy and quality of life moving forward.

## Discussion

The definite etiology of CHARGE syndrome has not yet been identified. However, the mutation in the CHD7 gene has been found in about two-thirds of CHARGE syndrome cases [7-9]. CHARGE syndrome is mainly caused by frameshift mutation or nonsense mutation de novo in the CHD7 gene [7]. The presence of this mutation results in protein haploinsufficiency that leads to multiple congenital diseases that are usually present in CHARGE syndrome [3]. Six congenital anomalies can be present in CHARGE syndrome, which include heart malformation, coloboma, choanal atresia, growth and development retardation, ear anomalies, and genital anomalies [10].

Our patient was suffering from several congenital anomalies, including developmental delay, coloboma, ear abnormalities, choanal atresia, micropenis, high arched palate, dilated left atrium, and a dilated left ventricle. Genitourinary problems due to CHARGE syndrome are easily recognized in males as micropenis or cryptorchidism [11]. On genetic investigation, he was found to have a mutation in the CDH7 gene, which can be found in up to 70% of patients with CHARGE syndrome and is considered confirmatory [5].

One of the hypotheses of why CHARGE syndrome occurs is that it can be due to some teratogenic agents received during pregnancy or increased paternal age [12]. However, the mother of the patient was 23 years old with no reported history of radiation exposure, smoking, illnesses, or any drugs during her pregnancy. Furthermore, CHARGE syndrome can be sporadic in the majority of cases, with no familial history [4,11]. On the other hand, there were a few familial cases that have been reported in the literature [13]. Hughes et al. reported that there can be some criterion of predisposition that can be related to the incidence of CHARGE syndrome such as a family history of clefting [14]. However, in our case, the patient’s family had no history of CHARGE syndrome or any genetic diseases, as well as no history of clefting, making it a sporadic type.

Breathing obstruction at birth is the result of choanal atresia, which was reported in 65% of cases and is usually corrected surgically to improve the patient’s survivability and life expectancy [11]. It was reported that infants with CHARGE syndrome are usually born with normal birth weight, and even though the majority of CHARGE syndrome patients experience poor growth during late infancy, they are usually born within the range of normal birth weight [11]. A previous case report also reported a baby girl with CHARGE syndrome who had a normal birth weight of 2.86 kg, similar to our case but was also suffering from choanal atresia, coloboma, and ear anomalies [15].

The management of CHARGE syndrome requires a multidisciplinary approach and several surgical interventions to improve the quality of life and improve the life expectancy of the patient [4]. Some studies reported some patients with CHARGE syndrome marking 30 years of age with the right multidisciplinary approach [16]. In our case, surgical correction of his choanal atresia and palate was performed to improve his breathing quality of life with a plan to wean him slowly from the nasal cannula oxygenation. Furthermore, close monitoring of his symptoms and systems in the next couple of years until the age of five is needed, as the first five years of life have been reported to have the highest mortality rate in CHARGE syndrome [17].

## Conclusions

CHARGE syndrome is a rare syndrome that usually occurs due to CDH7 gene mutation. Some other factors can also be linked with it such as exposure to teratogenic agents during pregnancy. In our case, the patient had a sporadic occurrence of the CDH7 gene mutation, which is considered the cause of CHARGE syndrome, as his mother was not exposed to any teratogenic materials nor did she have a previous illness. To increase the life expectancy of these patients, a multidisciplinary approach is crucial for passing the dangerous first five years of life.

## References

[1] Hall BD (1979). Choanal atresia and associated multiple anomalies. J Pediatr.

[2] Hittner HM, Hirsch NJ, Kreh GM, Rudolph AJ (1979). Colobomatous microphthalmia, heart disease, hearing loss, and mental retardation--a syndrome. J Pediatr Ophthalmol Strabismus.

[3] Pramudita JJ, Utari A, Winarni TI, Faradz SM (2017). CHARGE syndrome: an Indonesian case report. J Biomed Transl Res.

[4] Turkmen KA, Yildirim A, Oner CN (2013). A 2-month-old baby with CHARGE syndrome. Marmara Med J.

[5] Blake KD, Prasad C (2006). CHARGE syndrome. Orphanet J Rare Dis.

[6] Issekutz KA, Graham Jr JM, Prasad C (2005). An epidemiological analysis of CHARGE syndrome: preliminary results from a Canadian study. Am J Med Genet A.

[7] Aramaki M, Udaka T, Kosaki R (2006). Phenotypic spectrum of CHARGE syndrome with CHD7 mutations. J Pediatr.

[8] Janssen N, Bergman JE, Swertz MA (2012). Mutation update on the CHD7 gene involved in CHARGE syndrome. Hum Mutat.

[9] Lalani SR, Safiullah AM, Fernbach SD (2006). Spectrum of CHD7 mutations in 110 individuals with CHARGE syndrome and genotype-phenotype correlation. Am J Hum Genet.

[10] Essabar L, Aglili F, Barkat A (2018). An unusual case of CHARGE syndrome presenting with intrathoracic kidney and right-sided diaphragmatic hernia. Pediatric Oncall Journal.

[11] Hsu P, Ma A, Wilson M, Williams G, Curotta J, Munns CF, Mehr S (2014). CHARGE syndrome: a review. J Paediatr Child Health.

[12] Blake KD, Davenport SL, Hall BD (1998). CHARGE association: an update and review for the primary pediatrician. Clin Pediatr.

[13] Bergman JE, Janssen N, Hoefsloot LH, Jongmans MCJ, Hofstra RMW, van Ravenswaaij-Arts CMA (2011). CHD7 mutations and CHARGE syndrome: the clinical implications of an expanding phenotype. J Med Genet.

[14] Hughes SS, Welsh HI, Safina NP, Bejaoui K, Ardinger HH (2014). Family history and clefting as major criteria for CHARGE syndrome. Am J Med Genet A.

[15] Alshdefat A, Al-Mandhari H, Baker RA (2020). CHARGE syndrome (congenital anomalies): a case report. Drug Invent Today.

[16] Searle LC, Graham JM Jr, Prasad C, Blake KD (2005). CHARGE syndrome from birth to adulthood: an individual reported on from 0 to 33 years. Am J Med Genet A.

[17] Bergman JE, Blake KD, Bakker MK, du Marchie Sarvaas GJ, Free RH, van Ravenswaaij-Arts CMA (2010). Death in CHARGE syndrome after the neonatal period. Clin Genet.

